# The health impact of residential retreats: a systematic review

**DOI:** 10.1186/s12906-017-2078-4

**Published:** 2018-01-10

**Authors:** Dhevaksha Naidoo, Adrian Schembri, Marc Cohen

**Affiliations:** 1 School of Health and Biomedical Sciences, RMIT University, Plenty Rd, Bundoora, Bundoora, VIC 3083 Australia; 2 Cogstate Limited, Melbourne, 3000 Australia

**Keywords:** Wellbeing, Wellness tourism, Medical tourism, Lifestyle, Retreat experience, Multiple sclerosis, Cancer, Heart disease, Mental health

## Abstract

**Background:**

Unhealthy lifestyles are a major factor in the development and exacerbation of many chronic diseases. Improving lifestyles though immersive residential experiences that promote healthy behaviours is a focus of the health retreat industry. This systematic review aims to identify and explore published studies on the health, wellbeing and economic impact of retreat experiences.

**Methods:**

MEDLINE, CINAHL and PsychINFO databases were searched for residential retreat studies in English published prior to February 2017. Studies were included if they were written in English, involved an intervention program in a residential setting of one or more nights, and included before-and-after data related to the health of participants. Studies that did not meet the above criteria or contained only descriptive data from interviews or case studies were excluded.

**Results:**

A total of 23 studies including eight randomised controlled trials, six non-randomised controlled trials and nine longitudinal cohort studies met the inclusion criteria. These studies included a total of 2592 participants from diverse geographical and demographic populations and a great heterogeneity of outcome measures, with seven studies examining objective outcomes such as blood pressure or biological makers of disease, and 16 studies examining subjective outcomes that mostly involved self-reported questionnaires on psychological and spiritual measures. All studies reported post-retreat health benefits ranging from immediately after to five-years post-retreat. Study populations varied widely and most studies had small sample sizes, poorly described methodology and little follow-up data, and no studies reported on health economic outcomes or adverse effects, making it difficult to make definite conclusions about specific conditions, safety or return on investment.

**Conclusions:**

Health retreat experiences appear to have health benefits that include benefits for people with chronic diseases such as multiple sclerosis, various cancers, HIV/AIDS, heart conditions and mental health. Future research with larger numbers of subjects and longer follow-up periods are needed to investigate the health impact of different retreat experiences and the clinical populations most likely to benefit. Further studies are also needed to determine the economic benefits of retreat experiences for individuals, as well as for businesses, health insurers and policy makers.

## Background

Lifestyle-related chronic diseases such as obesity, diabetes and lung disease are a global issue, which the World Health Organisation estimate account for 60% of all deaths [1]. These diseases are characterised by modifiable risk factors such as physical inactivity, unhealthy diet such as diets high in salt, sugar, fat, alcohol and tobacco, and exposure to environmental toxicants [1]. Unhealthy lifestyles are a major factor in the development of chronic disease and are directly addressed by the health retreat industry, which promises to deliver enhanced health and the reversal of chronic disease and age-related conditions by engaging people directly in healthy lifestyle behaviours and experiences [2–4].

Health retreats have emerged from a history of travel to foreign destinations such as spas, hot springs, sacred sites, and pilgrimage locations that have been used as places of rest and rejuvenation for countless generations [3, 5]. Such locations have given rise to a booming wellness tourism industry that is estimated to have generated US$563.2 billion in revenues in 2015, with growth projections that are nearly 50% faster than for overall global tourism [6, 7]. A retreat may be defined as “a purpose-built centre which accommodates its guests for the purpose of learning/improving a body-mind activity (e.g., yoga, pilates) and/or learning-receiving complementary therapies or treatments whilst there” [2]. Retreats cover a broad spectrum of facilities ranging from low-cost ashrams in India [2, 3, 8] that focus on a spiritual-based lifestyle, to luxury lifestyle resorts [8], to residential centers that focus on chronic disease. Retreat guests range from people who want to improve their general health and learn positive lifestyle practices, to those facing life-threatening illnesses, and others who seek greater spiritual awareness or body-mind-spirit rejuvenation [9].

Despite the growing popularity of retreats and the growth of wellness tourism, the health impacts (either positive or negative) of attending retreats are uncertain and it is unclear if retreats offer any economic return for individuals or other stakeholders such as businesses, health insurance companies or governments. This paper aims to systematically review published studies that report on the health, wellbeing and economic impact of retreat experiences according to the PICOS approach (Participants, Intervention, Comparators, Outcomes and Study design) [10], and thereby explore the impact of these experiences on retreat guests.

## Methods

### Search strategy and data sources

A literature search was conducted in February 2017 using the electronic databases MEDLINE, CINAHL, and PsychINFO using search terms as appropriate for each of the databases. The search used combinations of keywords and phrases (retreat, health, wellness, wellbeing, resident) to conduct the systematic review. Truncation of keywords was used where variations of these words may alter search results. In addition reference lists of all relevant studies were manually searched.

### Inclusion/ exclusion criteria

Studies were included if they were published prior to February 2017, written in English, and contained before and after data related to health and wellbeing of retreat guests. As there is no strict definition of ‘retreat’, we included all studies that had at least one health-related outcome and the intervention involved a residential setting of one or more nights. Studies that did not meet the above criteria or contained only descriptive data from interviews or case studies were excluded. We did not exclude studies based on the purpose of the retreat.

### Data extraction

Each potentially eligible study identified in the literature search was independently screened according to the study inclusion criteria and then independently reviewed. Detailed summary tables of the studies were prepared according to the PICOS approach [10]. Participants included both healthy people and people with specific diseases who attended the relevant retreat program; the interventions were all residential retreat programs that involved one or more nights stay excluding hospital stays; comparisons were made between post-retreat and pre-retreat measures; and outcomes included any physiological, psychological, or other clinically relevant outcomes. Data from all included studies were extracted by two independent authors and presented in Tables 1, 2 and 3 along with *p* values when p was less than 0.05.

**Table 1:** Summary of Randomised Controlled Trials of Retreat Interventions

Reference	Study design	Population (includes comparator group/s)	Intervention	Place (bold font indicates country)	Comparator	Timing of measures	Outcome Measures	Results
Epel et al., 2016 [1]	RCT	Healthy women (*n* = 94); Experienced meditator (*n* = 30), Novice meditator (*n* = 33), Vacation (*n* = 31)	5-day Meditation Retreat: Meditaton and yoga.	Chopra Center for Wellbeing, La Costa Resort, Carlsbad, California, **United States**	Vacation at the same venue without meditation retreat activities	Pre-retreat, post-retreat, 1 and 10 months post-retreat.	Gene expression changes (transcriptome-wide expression patterns), aging-related biomarkers (telomerase activity, Aβ peptide levels), depressive symptoms, perceived stress, vitality and mindfulness.	Highly sig. Gene expression changes observed across all groups post-retreat (the ‘vacation effect’) characterized by improved regulation of stress response, immune function and amyloid beta (Aβ) metabolism. Sig. improvement in all groups in depressive symptoms, perceived stress, mindful awareness and vitality immediately after and 1-month post-retreat. The novice group improved sig more on mindfulness than the other two groups at day 5 and at 1-month and 10-months post-retreat.
Mills et al., 2016 [2]	Quasi-randomised trial	Healthy men and women (*n* = 119); Intervention (*n* = 65), Vacation ((*n* = 54)	6-day Panchakarma Ayurvedic Retreat ‘Perfect Health (PH) Program’: Physical cleansing through ingestion of herbs, fiber, and oils. Twice-daily Ayurvedic light plant-based meals. Daily Ayurvedic oil massage, heating treatments (sauna and/or steam, lectures on Ayurvedic principles, lifestyle, meditation and yoga philosophy. Twice-daily group meditation, daily yoga and breathing exercises (pranayama), emotional expression through journaling and emotional support. Integrative medical consultation (1-h) with a physician and follow-up with Ayurvedic health educator.	Chopra Center for Wellbeing, La Costa Resort, Carlsbad, California, **United States**	Vacation at the same venue without meditation retreat activities	Pre-retreat, post-retreat and 1 and 10 months post-retreat.	The Spirituality Scale, Gratitude questionnaire, Self-Compassion Scale, Ryff Scale of Psychological Wellbeing, Center for Epidemiology Studies-Depression (CES-D) tool, Patient-Reported Outcomes Measurement System (PROMIS) Anxiety Scale. *Other outcomes obtained were BP, height, weight (reported in Peterson 2016)*	Sig. increases in spirituality (*p* < 0.01) and gratitude (*p* < 0.05) in the retreat group and no change in control group. Sustained increases in spirituality (*p* < 0.01), gratitude. and self-compassion (*p* < 0.01) and reduced anxiety (*p* < 0.05) at 1-month follow-up.
Peterson et al., 2016 [3]	RCT	Healthy men and women (*n* = 119); Intervention (*n* = 65), Vacation (*n* = 54)	6-day Panchakarma Ayurvedic Retreat ‘Perfect Health (PH) Program’: Ayurvedic herbs using the Zrii purify herbal program, vegetarian diet, meditation, yoga, Ayurvedic oil massage, heat therapies and lectures on self-care and wellbeing.	Chopra Centre for Wellbeing, La Costa Resort, Carlsbad, California, **United States**	Vacation at the same venue without meditation retreat activities	Pre and post-retreat	BMI, systolic and diastolic BP, heart rate, saliva, stool, fasting blood sample, alcohol use, caffeine use, biological markers of cell biology, genome, metabolome and microbiome. *Psychological indices of wellbeing (reported in Mills 2016)*	Statistically sig. Changes (decrease) in plasma levels of phosphatidylcholines, sphingomyelins and others after 6 days.
Taren et al., 2015 [4]	Single-blind RCT	Stressed unemployed job-seeking community adults ((*n* = 35); Intervention (*n* = 18), Vacation (*n* = 17)	3-day Enhancement Through Mindfulness (HEM) Retreat: Mindfulness training through body scan awareness exercises, sitting and walking meditations, mindful eating and mindful movement (gentle hatha yoga postures), discussion of individual observations and practices.	Residential Retreat, Pittburgh, Pennsylvania, **United States**	Vacation at the same venue without meditation retreat activities	Pre-retreat (up to 4 weeks before), post-retreat (up to 2 weeks after) and 4 months post-retreat	Neuroimaging assessment (resting state functional connectivity (rsFC) scan), hair sample (cumulative hypothalamic-pituitary-adrenal (HPA) axis activation), Perceived Stress Scale (PSS).	Sig. changes in resting state functional connectivity (rsFC) in the right amygdala-subgenual anterior cingulate cortex (sgACC) of intervention group (time-treatment interaction *p* < 0.05).
Gilbert et al., 2014 [5]	RCT	Women, aged 31 to 60, with no meditation experience (*n* = 66) randomised to intervention or vacation ((*n* = not reported) control (n = not reported)	5-day resort stay to attend meditation, yoga, awareness and self-reflection training (intervention) or to relax at the resort and receive health lectures (control). Both groups received the same diet.	Chopra Center for Wellbeing, La Costa Resort, Carlsbad, California, **United States**	Vacation at the same venue without meditation retreat activities	Pre- and post-retreat	Stress, affect, reactivity and rumination (end-of day diaries).	Sig. increase in positive affect and decrease in negative affect post-retreat in the retreat but not the control group. Both groups felt less ‘stressed’ post-retreat (p’s < .001).Only retreat women reported sig. Greater control over stressors (*p* = .01). All participants reported decreased rumination post-retreat, with more pronounced changes in the retreat group (p’s < .001).
Pidgeon et al., 2014 [6]	RCT	Human services professionals (*n* = 44); Intervention (*n* = 22), Nil intervention ((*n* = 22)	2.5-day Mindfulness with Metta Training (MMTP) Retreat and 2 × 4-Hour Follow-up over 12-weeks: Periods of silence, training in mindfulness and metta skills and cognitive therapy strategies to increase mindfulness and self-compassion. Follow-up included review and practice of mindfulness, metta and cognitive strategies.	Residential Facility, Southern Queensland, **Australia**	No intervention	Pre-retreat, post-retreat, 1 and 4 months post-retreat	Resilience (The Resilience Scale), Mindfulness (The Five Facet Mindfulness Questionnaire) and Self-compassion (The Self-Compassion Scale).	No sig. Differences reported immediately post-retreat with sig. Improvements in mindfulness and self-compassion in the retreat group at 1- and 4-months post-retreat and in resilience at 4-months post-retreat.
Kwiatkowski et al., 2013 [7]	Randomised multicenter trial	Non-metastatic breast cancer patients in complete remission (*n* = 232) intervention (*n* = 117), control (*n* = 115)	13-day SPA stay by small groups of patients comprising physical training, dietary education, physiotherapy and SPA cares.	Three SPA centres: Vichy, Le-Mont-Dore, Chaˆtel-Guyon, **France**	Not reported	Pre-retreat and every 6 months post-retreat for next 3 years	Anthropometric measures; Quality of Life (SF36 questionnaire), Anxiety and Depression (Hospital Anxiety and Depression (HAD) questionnaire)	Sig. increase in SF36 score by 9.5 points (*p* < 0.001), 4.6 (*p* < 0.5) and 6.2 (p < 0.05) respectively at 6, 12 and 24 months. Anxiety and depression score were reduced at 6, 12 and 24 months.
Brazier et al., 2006 [8]	RCT	HIV/ AIDS patients (*n* = 47); Intervention (*n* = 20), Standard care (*n* = 27)	15-day Art-of-Living with HIV Retreat and Weekly Follow-up for 12 weeks: Breathing techniques, meditation, movement and group process. Three breathing exercises are the essential elements of the program, particularly the Sudarshan Kriya or Healing Breath. At the end of the retreat, participants were given a daily home practice. Follow-up sessions included reviewing procedures from retreat.	Residential AOL facility in Quebec, standard care and follow-up in Vancouver, **Canada**	Standard care	Pre-retreat and 1, 6 and 12 weeks post retreat	General well-being, Mental Health Index (MHI), Health-related quality of life (MOS-HIV Survey), Stress (Daily Stress Inventory (DSI)).	Sig. positive changes in wellbeing, post-retreat with no change at later time points.

**Table 2:** Summary of Non-Randomised Controlled Trials of Retreat Interventions

Reference	Study design	Population (includes comparator group/s)	Intervention	Place (bold font indicates country)	Comparator	Timing of measures	Outcome Measures	Results
Al-Hussaini et al., 2001 [9]	Observational study with control	Vipassana Meditation Course participants (*n* = 45); Intervention (*n* = 14), Nil Intervention (*n* = 31)	10-day Vipassana Meditation retreat involving silent sitting and/or walking meditation, avoidance of caffeine and alcohol, specific breathing practices and daily lectures.	Muscat, **Oman**	No intervention	Pre and post-retreat	General Health (General Health Questionnaire (GHQ-28))	Sig. improvements in physical and psychological well-being in the Vipassana but not control group.
Khurana & Dhar, 2000 [10]	Observational study with controls	Male and Female Prison inmates (*n* = 150); Intervention (*n* = 75), Nil Intervention (*n* = 75).	10-day Vipassana Meditation retreat involving silent sitting and/or walking meditation, avoidance of caffeine and alcohol, specific breathing practices and daily lectures.	Tihar Jail, **India**	No intervention	Pre and post-retreat	Subjective Well-being, scale), Quality of Life (Life Satisfaction Scale), Criminal Propensity Scale.	Sig. improvements in Criminal Propensity and Subjective Well-being in male inmates of Vipassana group compared with conrol.
Emavardhana & Tori, 1997 [11]	Observational study with controls	Teenagers, some teachers and other adults (*n* = 719); Intervention (*n* = 438), Nil Intervention (*n* = 281)	7-day Vipassana Meditation retreat involving silent sitting and/or walking meditation, avoidance of caffeine and alcohol, specific breathing practices and daily lectures.	Young Buddhists Association Retreat Center, Bangkok, **Thailand**	No intervention	Pre and post-retreat	Self Esteem (Tennessee Self-Concept Scale (TSCS)), Life Style Index, Buddhist Beliefs and Practices Scale	Sig. improvement in self-esteem and self-concept post-retreat
Chandiramani et al., 1995 [12]	Observational study (study I) Observational study with control (study II)	Prison inmates (*n* = 270); Study I (*n* = 120), No comparator. Study II (*n* = 150), Intervention (*n* = 85), Nil Intervention (*n* = 65).	10-day Vipassana Meditation retreat involving silent sitting and/or walking meditation, avoidance of caffeine and alcohol, specific breathing practices and daily lectures.	Tihar Jail, **India**	No intervention	Pre-retreat, post-retreat, 3 and 6 months post-retreat (study II only)	Well-being (Psychological General Well-being Index (PGI) scale), Hope (Miller and Power hope scale), hostility questionnaire	Sig. improvement in physical and psychological health in the intervention group (Study II). Both studies showed sig. Reductions in anxiety and depression scores post-retreat (p < 0.001) in the Vipassana group but not in the control group.
Garland et al., 2009 [13]; Garland, 2007 [14]; Angen et al., 2002 [15]	Longitudinal cohort study with control	Advanced breast, prostate or colon cancer patients (*n* = 15), their partners (*n* = 15), natural history group of patients (n = 20) and their partners (*n* = 20)	5-day Tapestry Psychosocial Retreat: Intensive psychosocial intervention for palliative care patients and their partners based on the Commonweal Cancer Help Program.	Retreat and Renewal Centre outside of Calgary, **Canada**	No intervention	Pre-retreat, post-retreat, 1, 3, 6, 9, and 12 months post-retreat	Quality of Life (Functional Assessment of Cancer Therapy – General Form (FACT-G), McGill Quality of Life Questionnaire (MQOL), Quality of Life in Life Thretreating Illness – Family (QOLLTI-F) questionnaire, Fatigue (Functional Assessment of Cancer Therapy – Fatigue (FACT-F)), Spirituality and Purpose (Functional Assessment of Chronic Ilness and Treatment-Spirtuality Subscale FACIT-Sp)) Depression (Beck Depression Inventory-II, Hopelessness Scale, Brief Symptom Inventory-18), Index of marital satisfaction (IMS).	Patients in the tapestry group demonstrated Sig. improvement in marital satisfaction (*p* = .011) with less psychological wellbeing (*p* = 0.029), support (*p* = 0.021) and poorer social wellbeing (*p* = 0.01)than patients in the natural history group. Partners of patients in the Tapestry group reported more financial worries *p* = 0.05, and less marital satisfaction *p* = 0.05 than partners of patients not attending the retreat. Both the Tapestry and natural history groups reported more fatigue as time progressed regardless of groups.
Ornish et al., 2013 [16]; Ornish et al., 2008 [17]	Descriptive study with control	Men with biopsy-proven low-risk prostate cancer (*n* = 35); Intervention (*n* = 10), Standard care (*n* = 25)	3-day Lifestyle Modification Retreat and Outpatient phase as part of 3-month Comprehensive Lifestyle Modification Program: Low-fat, wholefoods, plant-based diet with supplements. Stress management (gentle yoga-based stretching, breathing, meditation, imagery, and progressive relaxation), moderate aerobic exercise and weekly group support sessions. Education and counselling by registered dietitian, exercise physiologist, clinical psychologist, nurse, and stress management instructor. Outpatient phase included weekly telephone contact with a study nurse.	Retreat location not reported, **United States**	Standard care	Pre-retreat, post-retreat and 5 years post-retreat	BMI, blood pressure, relative telomere length of peripheral blood mononuclear cells and telomerase activity, Lifestyle adherence (Lifestyle-index scores).	Sig. improvements in weight, abdominal obesity, blood pressure, and lipid profile were observed (all P < 0.05). Sig. increase in relative telomere length after 5 years in retreat group compared to decrease in control. Adherence to lifestyle changes associated with sig. Increase in telomere length compared with control.

**Table 3:** Summary of Longitudinal Cohort Studies of Retreat Interventions

Reference	Study design	Population (includes comparator group/s)	Intervention	Place (bold font indicates country)	Timing of measures	Outcome Measures	Results
Newberg et al., 2017 [18]	Observational study	Christian faith (*n* = 14), No comparator	7-day Ignatian Spiritual Retreat: Morning mass, personal reflection, contemplation, prayer, daily meeting with Spiritual Director. Meals eaten in common dining area with other retreatants but typically maintain overall silence of the retreat.	Jesuit Center, Wernersville, Pennsylvania, **United States**	Pre-retreat (up to 1 month before) and post-retreat (up to 2 weeks after)	Dopamine and seratonin transporter binding in midbrain (DaTscan single photon emission computed tomography (SPECT)), Speilberger State Trait Anxiety Inventory (STAI-Y), Profile of Moods Scale (POMS), Beck Depression Inventory (BDI), Short Form Health Survey (SF-12), Cloninger Self Transcendence Scale, Spirituality (Index of Core Spiritual Experiences (INSPIRIT))	Sig. decreases in dopamine transporter binding in the basal ganglia and in serotonin transporter binding in the midbrain post-retreat. Sig. changes in a variety of psychological and spiritual measures including improvement in perceived physical health, decreases in tension and fatigue, more intense religious and spiritual beliefs, feeling more religious and more spiritual and increase in feelings of self-transcendence.
Cohen et al., 2017 [19]	Observational study	Gwinganna Lifestyle retreat guests (*n* = 37), No comparator	7-day Gwinganna Lifestyle Retreat: Choice of nature walks, boxing, dance, spin classes, qi gong, yoga, Pilates, meditation, educational talks, spa treatments, massage, body treatments, counseling sessions, and other healing modalities. Organic diet with mainly plant-based foods, some fish and egg protein, no added sugar or salt, no gluten, dairy, caffeine, alcohol, red meat, or canned or packaged food.	Gwinganna Lifestyle Retreat, Tallebudgera Valley, Queensland, **Australia**	Pre-retreat, post-retreat, 6 weeks post-retreat.	Height, weight, abdominal girth, blood pressure, urinary pesticide metabolites; food and health symptom questionnaire, Five Factor Wellness Inventory (FFW), Pittsburgh Insomnia Rating Scale (PIRS), Depression, Anxiety Stress Scales (DASS), Profile of Mood States (POMS), Generalized Self-Efficacy Scale (GSE), Health Symptom Questionnaire (HSQ), and Cogstate cognitive function test battery.	Sig. improvements in all anthropometric measures (*p* < 0.001) and psychological and health measures (*p* < 0.05) post-retreat with a trend for improved health symptom frequency and severity. Health symptom frequency and severity continued to improve and became statistically sig. 6-weeks post-retreat, other measures reduced somewhat and were no longer statistically sig., even though they remained below pre-retreat levels.
Steinhubl et al., 2015 [20]	Observational study	Experienced and novice meditators (*n* = 40); Experienced (*n* = 20), Novice (*n* = 20)	7-day Wellness retreat: Silent mantra meditation, talks, guided deep breathing exercise (pranayama), yoga and other activities supporting inner calm in individual and group settings.	Retreat location not reported, **United States**	Pre and post-retreat	Heart rate and heart rate variability (HRV), mean arterial pressure, electroencephalograph ((EEG); 14 sensors plus 2 references)	Sig., measureable EEG changes in experienced and novice meditators. Meditation was associated with a small, but statistically sig. Decrease in blood pressure in a normotensive population.
Hadgkiss et al., 2013a [21]; Li et al., 2010 [22].	Longitudinal cohort study	Multiple Sclerosis patients (*n* = 274); No comparator	5-day Lifestyle Modification Retreat: Low-fat, plant-based diet, exercise, sunlight exposure, vitamin D and omega-3 supplementation. Educational program, meditation and stress reduction techniques, counselling, yoga and qigong.	The Gawler Foundation, Victoria, **Australia**	Pre-retreat and 1, 2.5 and 5 years post retreat (2.5 years phased out)	Health-related quality of life (HRQOL), Multiple Sclerosis Quality Of Life Questionnaire (MSQOL-54)	Sig. improvements in HRQOL including overall quality of life domain (*p* < 0.001); physical health composite (*p* < 0.001); and mental health composite (*p* < 0.001). Further improvements at 5 years for overall quality of life; physical health composite and mental health composite
Vella & Budd et al., 2011 [23]	Observational study	Female reast cancer patients (*n* = 28); No comparator	7-day Photographic Art Therapy Retreat: Photographic art therapy in concert with psychoanalytically oriented group therapy, mind-body practices (optional yoga and meditation), lectures, discussion and support groups and very low-fat diet and exercise.	F. Holland Day Center for Creativity and Healing, Georgetown, Maine, **United States**	Pre-retreat, post-retreat and 6 weeks post-retreat	Anxiety, depression, and somatic symptoms (Brief Symptom Inventory-18 (BSI)), Quality of life (Functional Assessment of Cancer Therapy-General (FACT-G)), Spiritual well-being (Functional Assessment of Chronic Illness Therapy-Spiritual Well-being (FACIT-sp) subscale).	Sig. reductions in depression, anxiety and somatic stress and sig. Improvements in QoL and spiritual wellbeing that were sustained after 6 weeks.
Conboy et al., 2009 [24]	Observational study	Women (*n* = 20); No comparator	5-day Panchakarma Ayurvedic Retreat and 3-weeks (Min.) Pre-Retreat and 2-weeks Post-Retreat: Individual assessments, massage treatments, cleansing diet, yoga session, cooking class and group discussion. Pre-intervention includes guidance to modify diet and begin taking common herbal supplements. Post-intervention continues the cleansing process with lifestyle recommendations to maintain balance long term.	Kripalu Centre for Yoga and Health, **United States**	Pre-retreat, post-retreat and 3 months post-retreat	Health-Promoting Lifestyle Profile, Quality of life (SF-12), Self efficacy (single measure), Anxiety (Beck Anxiety Inventory), Social support (Interpersonal Support Evaluation List and Sarason Scoial Support Questionnaire), Perceived Stress Scale).	Sig. improvements in self-efficacy towards using Ayurveda to improve health with sig. Improvements in perceived social support and depression 3 months post-retreat
Kennedy et al., 2003 [25]	Observational study	Rice Diet Program Participants (*n* = 101); No comparator	10-day (Min.) Rice Diet Retreat: Very low-fat diet and exercise. Optional participation in yoga and meditation classes. Lectures, discussion and support groups, including a discussion group on spirituality.	Durham, North Carolina, **United States**	Pre and post-retreat	Spirituality (3 item questionnaire), well-being (12 item questionnaire), meaning in life (1 item questionnaire) and anger (4 item questionnaire).	Increased spirituality positively associated with increased well-being, increased sense of meaning and purpose in life, and decreased tendency to become angry.
Beatus et al., 2002 [26]	Observational study	People with Multiple Sclerosis (*n* = 41)	6-day summer retreat offered annually by The Multiple Sclerosis Society to individuals with MS. The retreat encourages physical activity, art, and social interaction.	Specific location not stated, **United States**	Pre- and post-retreat	Rosenburg Self-Esteem Scale (Self-E), Multiple Sclerosis Quality of Life-54 Instrument (MSQOL-54), Activities of Daily Living (Activities of Daily Living Self Care Scale for persons with multiple sclorisis (ADL-MS).	Sig. increase in the mental component of quality of life.
Kennedy et al., 2002 [27]	Observational study	Patients with coronary disease and their partners (*n* = 72); Patients (*n* = 51), Partners (*n* = 21)	2.5-day Educational Retreat ‘Choice to Review’: Open discussions with healthcare professionals, activities such as stress-reduction techniques, (progressive relaxation, yoga, breathing exercises, visualization, and imagery), exercise options, nutritional counseling and vegetarian food, group exercises that encourage self-efficacy, enhance social support, build self-esteem and improve communication skills, and spiritual principles and techniques for healing (meditation, prayer and forgivesness)	Remote location, **United States**	Pre-retreat, post-retreat and 4–6 months post-retreat	Spirituality (3 item questionnaire), well-being (12 item questionnaire), meaning in life (1 item questionnaire) and anger (4 item questionnaire).	Changes in spirituality were positively associated with increased well-being, meaning in life, and confidence in handling problems, and with decreased tendency to become angry. No sig. Differences 4 and 6 months post-retreat.

### Risk of bias assessment

Two review authors independently assessed the risk of bias of each included Randomised Controlled Trial study using the Cochrane Risk of Bias tool including key criteria such as random sequence generation, allocation concealment, blinding of participants, blinding of personnel and outcomes, incomplete outcome data, selective outcome reporting, and other sources of bias in accordance with methods recommended by The Cochrane Collaboration [11]. The following judgements were used; low risk, high risk or unclear (either lack of information or uncertainty over poteintial for bias). Non-Randomised Controlled Studies and Longitudinal Cohort Studies were assessed using the Risk of Bias in Non-Randomised Studies-of Interventions (ROBINS-I) tool. Key criteria included confounding, participant selection, intervention classification, deviations from intended interventions, missing data, outcomes measurement and reported results. The following judgements were used; low risk, moderate risk, serious risk, critical risk or no information. Authors resolved disagreements by consensus, and a third author was available for consultation to resolve any discrepancies if necessary. Risk of bias assessments are summarised in Tables 4, 5 and 6.

**Table 4:** Risk of bias summary for Randomised Controlled Trial Studies

	Random sequence generation (selection bias)	Allocation concealment (selection bias)	Blinding of participants and personnel (performance bias)	Blinding of outcome assessment (detection bias) Self-reported outcomes	Incomplete outcome data (attrition bias)	Selective reporting (reporting bias)	Other bias
Epel et al., 2016 [1]	Low	Unclear	High	High	Low	Unclear	Low
Mills et al., 2016 [2]	High	High	High	High	High	Unclear	Low
Peterson et al., 2016 [3]	High	High	High	Low	Low	Unclear	Low
Taren et al., 2015 [4]	Low	High	High	High	Low	Unclear	Low
Gilbert et al., 2014 [5]	Low	Unclear	Unclear	High	Unclear	Unclear	Low
Pidgeon et al., 2014 [6]	Low	Unclear	Unclear	High	High	Unclear	Low
Kwiatkowski et al., 2013 [7]	Low	Unclear	Unclear	High	Low	Unclear	Low
Brazier et al., 2006 [8] [6]	Low	Unclear	Unclear	High	Unclear	Unclear	Low

**Table 5:** Risk of bias summary for Non-Randomised Controlled Trial Studies

	Bias due to confounding	Bias in selection of participants into the study	Bias in classification of interventions	Bias due to deviations from intended interventions	Bias due to missing data	Bias in measurement of outcomes	Bias in selection of the reported result
Al-Hussaini et al., 2001 [9]	Low	Low	Low	Low	Low	Moderate	Low
Khurana & Dhar, 2000 [10]	Low	Low	Low	Low	Low	Moderate	Low
Emavardhana & Tori, 1997 [11]	Low	Low	Low	Low	Low	Moderate	Low
Chandiramani et al., 1995 [12]	Low	Low	Low	Low	Low	Moderate	Low
Garland et al., 2009 [13]; Garland, 2007 [14]; Angen et al., 2002 [15]	Low	Low	Low	Low	Low	Moderate	Low
Ornish et al., 2013 [16]; Ornish et al., 2008 [17]	Low	Low	Low	Low	Low	Low	Low

**Table 6:** Risk of bias summary for Longitudinal Cohort Studies

	Bias due to confounding	Bias in selection of participants into the study	Bias in classification of interventions	Bias due to deviations from intended interventions	Bias due to missing data	Bias in measurement of outcomes	Bias in selection of the reported result
Newberg et al., 2017 [18]	Low	Low	Low	Low	Low	Moderate	Low
Cohen et al., 2017 [19]	Low	Low	Low	Low	Low	Moderate	Low
Steinhubl et al., 2015 [20]	Low	Low	Low	Low	Low	Moderate	Low
Hadgkiss et al., 2013a [21]; Li et al., 2010 [22].	Low	Low	Low	Low	Low	Moderate	Low
Vella & Budd et al., 2011 [23]	Low	Low	Low	Low	Low	Moderate	Low
Conboy et al., 2009 [24]	Low	Low	Low	Low	Low	Moderate	Low
Kennedy et al., 2003 [25]	Low	Low	Low	Low	Low	Moderate	Low
Beatus et al., 2002 [26]	Low	Low	Low	Low	Low	Moderate	Low
Kennedy et al., 2002 [27]	Low	Low	Low	Low	Low	Moderate	Low

## Results

**Fig. 1:**
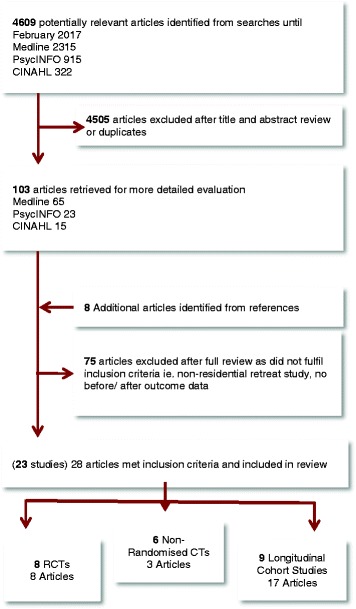
Study Flow Chart

There were 23 studies (reported in 28 articles) included in this systematic review, published over a 22-year period from 1995 to 2017 and involving 2592 participants. Of the 23 studies included, eight were randomised controlled trials (RCTs) including, one quasi-randomised trial and one randomised multi-centre trial; six non-randomised controlled trails and nine longitudinal cohort studies. A study flow chart is provided in Fig. 1. The results from the RCTs are presented in Table 1, results from the non-randomised controlled trials are presented in Table 2 and results from the longitudinal cohort studies are presented in Table 3.

### Participants

Studies in this review included a wide range of demographic and socioeconomic backgrounds including luxury resort guests [12–15], teachers [16], human service professionals [17], unemployed adults [18], and prison inmates [19, 20]. The reviewed studies also included participants with a wide range of health conditions. Eleven studies recruited participants in general health [12–15, 21–26], four studies recruited participants with mental health issues such as stress, fatigue or burnout [17–20], four studies recruited participants with cancers including prostate cancer, breast cancer and colon cancer [27–30], two studies recruited participants with multiple sclerosis [31–33], and the remaining two studies recruited participants with HIV/AIDS [34], and cardiac conditions [35].

The sample size in each study ranged from 14 [21] to 719 [16] with participants recruited from various locations including the local community and neighbouring areas [13–15, 17, 18, 21], specific medical facilities [28, 34, 36], prisons [19, 20], and secondary schools and colleges [16]. In four studies, participants were guests who had already registered to attend the retreat and were invited to participate in the research [12, 22, 24, 25]. Some studies did not report on how participants were recruited [23, 27, 31, 33, 35].

### Interventions

The retreat length of stays ranged from two and a half days [17, 35] to 15 days [34] with a duration of five to seven days being the most common [12–16, 21–23, 26, 28, 31–33, 36]. Four retreats included a follow-up intervention period [17, 23, 27, 30, 34] that ranged in frequency, duration and mode of delivery from a couple of four-hour sessions over 12 weeks [17] to weekly follow-up via telephone over a three-month period [27].

Retreat programs ranged from a focus on religion and spirituality to lifestyle, health and wellbeing. Spirituality-focused retreats involved different spiritual/religious denominations and practices including mindfulness meditation [13, 17, 18, 34], Vipassana meditation [16, 19, 20, 25], Ayurveda [14, 15] and Ignatian/Jesuit spirituality [21]. These retreats included activities such as prayer, mass, chanting, observing silence and other techniques such as breathing and mindfulness. Health and wellness-focused retreats included activities such as exercise, yoga, body treatments, medical consultations, counselling, support groups and discussion [12, 22, 27, 31, 33]. Both spiritually-focused and health-focused retreats commonly included meditation as an activity, sometimes optional, along with a focus on either a prescribed diet such as organic diet, [12] vegetarian diet [15, 35] or low-fat diet, [24, 27, 28, 31, 33] or dietary education such as mindful eating [16, 18] or nutritional counselling. [35] In the four studies with a follow-up intervention, activities included review and practice of techniques taught at the retreat such as mindfulness, [17, 34] the continuance of practices and processes that began at the retreat such as lifestyle changes and cleansing, [23] or telehealth support from a study nurse [27].

### Place

More than half of the studies (13) were conducted in the United States [13–15, 18, 21–24, 27, 28, 35], three studies were conducted in Australia [12, 17, 31, 33], two studies each were conducted in Canada [29, 34, 36] and India [19, 20], and the remaining three studies were conducted in Oman [25], Thailand [16] and France [30].

Studies were held at specifically designed retreat centres [34, 36], residential facilities such as religious centres [16, 21] or prisons [19, 20] as well as yoga [23] and healing retreat facilities [28]. Four studies were conducted with guests staying at luxury resorts, one in Queensland, Australia [12], and three at the same resort in California, United States [13–15]. Both studies conducted in India were conducted at a prison in New Delhi for prisoners [19, 20]. Three studies did not report the specific location of the retreat [18, 22, 27].

### Comparators

Of the eight controlled trials reviewed, five included vacation groups [13–15, 18, 26], who visited the same retreat purely for relaxation purposes without participation in organised retreat activities. One of these studies included an additional comparator group to compare results between novice and experienced meditators [13]. One study for HIV/AIDS patients had a group who continued to receive their standard care [34], and two studies; one for Human Service Professionals and another for non-metastatic breast cancer patients, had a group who received no intervention [17, 30].

Of the six non-randomised controlled trials reviewed, five studies included comparator groups who received no intervention [16, 19, 20, 25, 29] and one study included a group who received standard care [27]. Only two of the nine longitudinal cohort studies included a comparator group, with one study comparing results from novice to experienced meditators [22] and another comparing results from healthy heart patients to their partners [35].

### Outcome measures

All studies reported statistically significant improvements in at least one measured outcome at some time after retreat. Outcomes ranged from subjective measures using standardized self-reported questionnaires for wellbeing [14, 36], quality of life [19, 28–30, 36] and perceived stress [12, 13, 18, 23, 26, 28, 34] such as; The Gratitude, Resentment and Appreciation Test (GRAT-sf) [37, 38], The Ryff Scale of Psychological Wellbeing [39], and the Mental Health Index (MHI) [40]; to objective measures such as abdominal girth [12, 15, 27], blood pressure [12, 15, 22, 27] and analysis of urine [12], blood [13–15, 26, 27], hair samples [18], neuroimaging [18], cognitive function [12], gene expression [13] and the metabolome [15]. All studies included at least one before (pre-retreat) and one after (post-retreat) measurement with most studies including more than one post-retreat measurement, ranging from one month post-retreat [13, 14, 17, 36] to five years post-retreat [27, 31]. No studies reported any adverse effects or economic outcomes.

### Summary of objective and subjective outcomes

All studies reported statistically significant improvements in at least one measured outcome with only one study of 44 human service professionals undertaking a two-and-a-half day Mindfulness with Metta Training (MMTP) Retreat, reporting no significant differences in self-report measures of resilience, mindfulness and self-compassion immediately after the retreat experience, despite significant improvements for mindfulness and self-compassion at one and four months and for resilience at four months post-retreat [17]. A further study of 47 patients with HIV/AIDS who participated in a 15-day ‘Art-of-Living with HIV’ retreat reported significant positive changes in wellbeing immediately after the retreat, that were not evident after 6 and 12 weeks [34].

### Objective/ quantitative outcomes

All seven studies investigating objective outcomes reported statistically significant improvements immediately after the intervention. Three of these studies reported significant improvements in anthropometric measures such as weight, abdominal girth and blood pressure [12, 23, 27, 41] and reductions in blood lipids [41]. Statistically significant results were also reported for decreases in dopamine transporter binding in the basal ganglia and serotonin transporter binding in the midbrain [22]; changes in resting state functional connectivity (rsFC) in the right amygdala-subgenual anterior cingulate cortex (sgACC) [18]; reductions in 12 phosphatidylcholines and an additional 57 metabolites such as amino acids, biogenic amines, acylcarnitines, glycerophospholipids and sphingolipids [15]; gene expression changes associated with improved regulation of stress response, immune function and amyloid beta (Aβ) metabolism [13]; and electroencephalogram (EEG) changes [22]. Ornish et al. [27] further documented increases in relative telomere length after five years that was associated with the degree of adherence to lifestyle changes in ten of 35 men with biopsy-proven prostate cancer [27, 41].

### Subjective/ qualitative outcomes

Fifteen of the 16 studies investigating subjective or survey-based outcomes reported statistically-significant improvements immediately post-retreat including significant improvements in quality of life, perceived physical health and health symptoms, as well as a variety of psychological and spiritual measures [12–14, 16, 17, 19–21, 23, 25, 28, 29, 31, 34, 36]. Two studies reported improvements in overall health-related quality of life [28, 31] and four studies improvements in perceived physical health [20, 21, 25, 31]. Cohen et al. [12] reported improvements in both subjective and outcome measures including cognitive function and Conboy et al. [23] reported improvements in positive health behaviours and self-efficacy.

Eight of the nine studies measuring psychological wellbeing reported statistically significant improvements in a variety of indicators including depression, anxiety, tension, stress, fatigue, mindful awareness and vitality [12, 13, 20, 21, 25, 28, 29, 31, 36]. Khurana and Dhar [19] reported improvements in subjective wellbeing and criminal propensity, however this improvement was only seen in male inmates of the intervention group, and not in female inmates or the control group that did not receive the intervention. All six studies measuring spiritual wellbeing reported significant improvements in various religious and spiritual measures [14, 16, 21, 24, 28, 35]. Vella and Budd [28] reported improvements in overall spiritual wellbeing and Mills et al. [14] reported a significant increase in spirituality and gratitude in the intervention group that participated in a six-day Panchakarma Ayurvedic program, compared with no change in the control group that were on vacation at the same resort. Newberg et al. [21] reported significant changes such as more intense religious and spiritual beliefs, feeling more religious and more spiritual, and an increase in feelings of self-transcendence in 14 participants of a Christian faith.

Two studies [24, 35] investigating the relationship between spirituality and health measures, found that measures of spirituality increased after a retreat along with increased well-being, sense of meaning and purpose in life, confidence in handling problems and a decreased tendency to become angry. Similarly, Emavardhana and Tori [16] found that heightened belief in Buddhist precepts was associated with positive change in self-concept and less self-criticism and increased Buddhist religiosity was correlated with reductions in the defences of displacement, projection and regression [16].

### Risk of bias

All Randomised Controlled Trial Studies were found to have low, unclear or high risk of bias in one or more domains. The most high risk was reported for blinding of outcome assessment. Allocation concealment and reported data selection was not reported for the majority of studies and therefore unclear. All Non-Randomised Controlled Trial Studies and Longitudinal Cohort Studies were found to have low risk of bias for all domains except outcomes measures. All but one study [41] failed to report whether or not the outcomes assessor was aware of the participant intervention and were therefore found to have a moderate risk of bias for outcomes measurement. Given these findings, all studies are comparable to a well-performed randomised trial with regard to the majority of domains (low risk) except outcomes measurement (moderate risk) indicating the studies are sound for a non-randomised study with regard to this domain but cannot be considered comparable to a well-performed randomized trial,

## Discussion

The retreat industry is a niche sector of the wellness tourism industry that focuses on transformative experiences that aim to improve the health of participants through healthy lifestyle experiences, along with providing the skills and knowledge to help maintain healthy behaviours. The findings from the reviewed studies suggest there are many positive health benefits from retreat experiences that includes improvements in both subjective and objective measures. Most studies used a quasi-experimental design with small sample sizes, poorly described methodology with little follow-up data and reliance on self-report questionnaires to report on psychological and spiritual benefits. The results from the most rigorous studies that used randomized controlled designs were consistent with less rigorous studies and suggest that retreat experiences can produce benefits that include positive changes in metabolic and neurological pathways, loss of weight, blood pressure and abdominal girth, reduction in health symptoms and improvements in quality of life and subjective wellbeing.

In addition to facilitating general health improvements, there is evidence that retreat experiences can have a positive impact on chronic disease processes and provide benefits for some people with life threatening and/or chronic diseases. Of the four studies of retreat experiences aimed at improving quality of life for cancer patients [27, 28, 30, 36], all showed some benefits from retreat participation, including improvements in quality of life, depression and anxiety scores, and increased telomere length, with benefits being recorded up to five years post-retreat. Similarly, benefits of retreat participation are reported for people with multiple sclerosis with improvements in quality of life along with physical and mental health being evident up to five years post-retreat [31, 32]. Not all measures in the studies of life-threatening chronic diseases improved [30, 36], and as they are all small, poorly-controlled studies, more rigorous research is needed.

The finding that retreat experiences can lead to sustained and significant health improvements long after participants return home suggests that these experiences assist guests in making positive lifestyle changes and adopting healthy behaviours that lead to a variety of positive psychological, physiological, cognitive, clinical and metabolic effects. The ability to influence participants’ health once they return home is dependent on many factors including the type of participants involved, the education and experiences provided during the retreat program, and the provision of follow-up activities such as online coaching, nutrition programs, or follow-up consultations with practitioners. Of the four studies that showed a reduced effect over time in some measures [12, 17, 34, 35], two studies did not include a follow-up retreat component [12, 35].While it is not possible to determine which parts of the retreat intervention have the greatest influence, it is likely that improvements in health are due to a combination of psychological and behavioural factors that lead to better coping mechanisms and enhanced resilience to stress, as well as metabolic factors that lead to alterations in gene expression and DNA repair mechanisms that are evident in the observed changes in the metabolome [15] and teleomere length [27, 41].

Despite the potential for retreat experiences to benefit people with chronic and life threatening disease, the retreat industry does not routinely interact with the health care sector with few patients being referred to retreats by medical practitioners and retreat experiences generally not covered by third party payment schemes or eligible for tax deductions or incentives. The lack of integration between the healthcare and retreat sectors may be partly due to a lack of data with which to evaluate retreat experiences. Few retreats routinely collect and/or communicate data relevant to the healthcare sector, and even when formal studies such as those reviewed here are conducted, there is great heterogeneity in the range and scope of outcome measures, with few measures being comparable across studies. The retreat industry would therefore benefit from the use of a standardised dataset collected from guests on a routine basis. Such data could include a combination of psychological, cognitive, physiological, anthropometric and biochemical measures that together provide a holistic assessment of outcomes. This would allow retreat participants to evaluate and monitor the impact of their experiences and provide data to engage the medical profession and third party payers. It would also be beneficial for the industry to develop a standardised reporting system for retreat activities so that the influence of different types of retreat experiences can be assessed and results meaningfully compared across retreats and studies.

While retreat experiences appear to have positive health impacts, there is no published data on the economic impact of retreat experiences. There is however, substantial evidence that non-residential wellness programs, which share a similar focus on health promotion and lifestyle modification, provide a substantial economic return [42–44]. A review of 28 studies of corporate wellness programs [45] finds that the economic benefit of participation is substantially higher than the costs of providing the program. Stead [45] reports benefit-to-cost ratios averaging 3.4–1 which indicates that corporate companies receive on average US$3.40 for every US$1 invested in the respective wellness program. In addition to return on investment, employees benefit from participating in corporate wellness programs through experiencing better health, lowered disability payments and reduced health care expenditures, while companies benefit from reduced employee turnover, increased productivity [45] and reduced absenteeism and presenteeism along with intangible benefits such as being an employer of choice and attracting highly skilled employees and creating a positive corporate culture [45, 46].

While the economic benefits of corporate wellness programs are becoming well established, it is unclear if similar benefits are offered by residential retreats. Future studies that include a health economic analysis are therefore needed to determine the cost-benefits of retreat experiences and the return on investment for participants, businesses, health insurers and policy makers. This may enable retreat operators to advocate for tax benefits, as well as inclusion in health insurance policies, and corporate wellness schemes. Furthermore, there is no data on the occurrence of adverse events. Future studies would benefit from including measures of adverse outcomes to confirm the safety and efficacy of retreat interventions.

Despite the consistent reporting of positive health effects from retreat interventions across multiple study designs and locations, the ability to draw definitive conclusions for any one condition or population is limited due to poor methodological rigor and substantial heterogeneity in study design, length and type of retreat program, target population, outcome measures and length of follow-up. Furthermore, while the reviewed studies included subjects from a wide range of demographic groups in multiple countries, only published English language studies were reviewed and it is uncertain if the findings can be generalized to the wider population. The use of mostly self-selected populations also introduces the possibility of selection bias, while a lack of blinding and adequate controls may introduce performance bias due to exposure to factors other than the specific intervention such as the vacation effect whereby health can improve from simply being removed from normal routines and behaviours. The lack of any reported adverse events may further indicate reporting bias with researchers not actively looking to identify adverse outcomes, or outcome measurement tools not being designed to capture adverse outcomes. Future studies, with more rigorous methodology and long-term follow-ups are now needed to determine the longevity of any effects, their mechanisms of action and the conditions most likely to respond.

## Conclusion

The findings of this review suggest that retreat experiences appear to have positive health benefits that include benefits for people with chronic diseases. As the observed improvements in chronic diseases are based on a small number of patients, future research using larger numbers of subjects and longer follow-up periods is needed in order to determine the populations most likely to benefit and quantify any long-term health benefits. Future studies could also benefit from more rigorous study designs including the use of standardized outcome measures, more detailed descriptions of the retreat interventions and study population, and the inclusion of a health economics analysis in order to determine the economic benefits of retreat experiences for individuals, as well as for businesses, health insurers and policy makers.

## Abbreviations

Aβ: Amyloid Beta
BMI: Body Mass Index
BP: Blood Pressure
DNA: Deoxyribonucleic Acid
EEG: Electroencephalogram
GRAT-sf: The Gratitude, Resentment and Appreciation Test
HIV/AIDS: Human Immunodeficiency Virus and Acquired Immune Deficiency Syndrome
MHI: Mental Health Index
MS: Multiple Sclerosis
MTTP: Mindfulness with Meta Training Program
PICOS: Participant, Intervention, Comparators, Outcomes and Study Design)
PwMS: People with Multiple Sclerosis
RCT: Randomised Controlled Trial
RsFC: Resting state functional connectivity
SgACC: subgenual anterior cingulate cortex
WHO: World Health Organisation

## References

[1] World Health Organisation. Preventing Chronic Diseases. A Vital Investment: WHO Global Report., Geneva: World Health Organization. . 2005. www.who.int/chp/chronic_disease_report/en.

[2] Smith M, Kelly C (2006). Holistic tourism: journeys of the self?. Tour Recreat Res.

[3] Smith M (2003). Holistic holidays: tourism and the reconciliation of body, mind and Spirit. Tour Recreat Res.

[4] Smith M, Kelly C (2006). Wellness tourism. Tour Recreat Res.

[5] Gesler WM (1992). Therapeutic landscapes: medical issues in light of the new cultural geography. Social science &amp; medicine (1982).

[6] Yeung O, Johnston K. Global wellness economy monitor. Miami, Fl: Global Wellness Institute2017.

[7] Yeung O, Johnston K. The Global Wellness Tourism Economy. Miami, Fl: Global Wellness Institute 2014 March 1 2017.

[8] Lea J (2008). Retreating to nature: rethinking ‘therapeutic landscapes. Area.

[9] Kelly C (2012). Wellness tourism: retreat visitor motivations and experiences. Tour Recreat Res.

[10] Methley AM, Campbell S, Chew-Graham C, McNally R, Cheraghi-Sohi SPICO (2014). PICOS and SPIDER: a comparison study of specificity and sensitivity in three search tools for qualitative systematic reviews. BMC Health Serv Res.

[11] Higgins JP, Green S, editors. Cochrane Handbook for Systematic Reviews of Interventions Version 5.1.0 [updated March 2011]. The Cochrane Collaboration; 2011.

[12] Cohen MM, Elliott F, Oates L, Schembri A, Mantri N (2017). Do Wellness Tourists Get Well? An Observational Study of Multiple Dimensions of Health and Well-Being After a Week-Long Retreat. Journal of alternative and complementary medicine (New York, NY).

[13] Epel ES, Puterman E, Lin J, Blackburn EH, Lum PY, Beckmann ND (2016). Meditation and vacation effects have an impact on disease-associated molecular phenotypes. Transl Psychiatry.

[14] Mills PJ, Wilson KL, Pung MA, Weiss L, Patel S, Doraiswamy PM (2016). The self-directed biological transformation initiative and well-being. The Journal of Alternative and Complementary Medicine.

[15] Peterson CT, Lucas J, John-Williams LS, Thompson JW, Moseley MA, Patel S et al. Identification of altered Metabolomic profiles following a Panchakarma-based Ayurvedic intervention in healthy subjects: the self-directed biological transformation initiative (SBTI). Sci Rep 2016;6:32609. 10.1038/srep32609. http://www.nature.com/articles/srep32609 - supplementary-information. 10.1038/srep32609 PMC5017211 27611967

[16] Emavardhana T, Tori CD (1997). Changes in self-concept, ego defense mechanisms and religiosity following seven-day Vispassana meditation retreats. The Journal for the Scientific Study of Religion.

[17] Pidgeon AM, Ford L, Klaassen F (2014). Evaluating the effectiveness of enhancing resilience in human service professionals using a retreat-based mindfulness with Metta training program: a randomised control trial. Psychology, health & medicine.

[18] Taren AA, Gianaros PJ, Greco CM, Lindsay EK, Fairgrieve A, Brown KW (2015). Mindfulness meditation training alters stress-related amygdala resting state functional connectivity: a randomized controlled trial. Soc Cogn Affect Neurosci.

[19] Khurana A, Dhar PL (2000). Effect of Vipassana meditation on quality of life, subjective well-being, and criminal propensity among inmates of Tihar jail, Delhi.

[20] Chandiramani K, Verma SK, Dhar PL, and Agarwal N. Psychological effects of Vipassana on Tihar jail inmates: research report. Vipassana Research Institute 1995. http://www.vridhamma.org/Psychological-effects-on-tihar-jail-inmates.

[21] Newberg AB, Wintering N, Yaden DB, Zhong L, Bowen B, Averick N, et al. Effect of a one-week spiritual retreat on dopamine and serotonin transporter binding: a preliminary study. Religion, Brain & Behavior. 2017:1–14. 10.1080/2153599X.2016.1267035.

[22] Steinhubl SR, Wineinger NE, Patel S, Boeldt DL, Mackellar G, Porter V, et al. Cardiovascular and nervous system changes during meditation. Frontiers in Human Neuroscience. 2015, 9;(145) 10.3389/fnhum.2015.00145. 10.3389/fnhum.2015.00145 PMC4364161 25852526

[23] Conboy LA, Edshteyn I, Garivaltis H (2009). Ayurveda and Panchakarma: measuring the effects of a holistic health intervention. TheScientificWorldJOURNAL.

[24] Kennedy JE, Rosati KG, Spann LH, King AD, Neelon FA, Rosati RA. Changes in spirituality and well-being in a medically based lifestyle program. 2003. http://jeksite.org/research/riceup.pdf

[25] Al-Hussaini AA, Dorvlo ASS, Antony SX, Chavan D, Dave J, Purecha V (2001). Vipassana meditation:: A naturalistic, preliminary observation in Muscat. Journal for scientific research Medical sciences / Sultan Qaboos University.

[26] Gilbert A, Epel E, Tanzi R, Rearden R, Schilf S, Puterman E (2014). A Randomized Trial Comparing a Brief Meditation Retreat to a Vacation: Effects on Daily Well-Being. The Journal of Alternative and Complementary Medicine.

[27] Ornish D, Lin J, Chan JM, Epel E, Kemp C, Weidner G (2013). Effect of comprehensive lifestyle changes on telomerase activity and telomere length in men with biopsy-proven low-risk prostate cancer: 5-year follow-up of a descriptive pilot study. The Lancet Oncology.

[28] Vella EJ, Budd M (2011). Pilot study: retreat intervention predicts improved quality of life and reduced psychological distress among breast cancer patients. Complement Ther Clin Pract.

[29] Garland S, Carlson L, Marr H, Simpson S (2009). Recruitment and retention of palliative cancer patients and their partners participating in a longitudinal evaluation of a psychosocial retreat program. Palliative & Supportive Care.

[30] Kwiatkowski F, Mouret-Reynier MA, Duclos M, Leger-Enreille A, Bridon F, Hahn T (2013). Long term improved quality of life by a 2-week group physical and educational intervention shortly after breast cancer chemotherapy completion. Results of the ‘Programme of accompanying women after breast cancer treatment completion in thermal resorts’ (PACThe) randomised clinical trial of 251 patients. Eur J Cancer.

[31] Hadgkiss EJ, Jelinek GA, Weiland TJ, Rumbold G, Mackinlay CA, Gutbrod S (2013). Health-related quality of life outcomes at 1 and 5 years after a residential retreat promoting lifestyle modification for people with multiple sclerosis. Neurological sciences : official journal of the Italian Neurological Society and of the Italian Society of Clinical Neurophysiology.

[32] Beatus J, O'Neill JK, Townsend T, Robrecht K (2002). The effect of a one-week retreat on self-esteem, quality of life, and functional ability for persons with multiple sclerosis. J Neurol Phys Ther.

[33] Li MP, Jelinek GA, Weiland TJ, Mackinlay CA, Dye S, Gawler I (2010). Effect of a residential retreat promoting lifestyle modifications on health-related quality of life in people with multiple sclerosis. Qual Prim Care.

[34] Brazier A, Mulkins A, Verhoef M (2006). Evaluating a yogic breathing and meditation intervention for individuals living with HIV/AIDS. American journal of health promotion : AJHP.

[35] Kennedy JE, Abbott RA, Rosenberg BS (2002). Changes in spirituality and well-being in a retreat program for cardiac patients. Altern Ther Health Med.

[36] Garland S. A pilot project to assess the impact of a psychosocial retreat intervention on the quality of life, distress, marital satisfaction and existential concerns in palliative cancer patients and their partners: ProQuest Dissertations Publishing; 2007.

[37] Froh JJ, Fan J, Emmons RA, Bono G, Huebner ES, Watkins P (2011). Measuring gratitude in youth: assessing the psychometric properties of adult gratitude scales in children and adolescents. Psychol Assess.

[38] McCullough ME, Emmons RA, Tsang JA (2002). The grateful disposition: a conceptual and empirical topography. J Pers Soc Psychol.

[39] Ryff CD, Singer B (1996). Psychological well-being: meaning, measurement, and implications for psychotherapy research. Psychother Psychosom.

[40] Veit CT, Ware JE (1983). The structure of psychological distress and well-being in general populations. J Consult Clin Psychol.

[41] Ornish D, Magbanua MJM, Weidner G, Weinberg V, Kemp C, Green C (2008). Changes in prostate gene expression in men undergoing an intensive nutrition and lifestyle intervention. Proc Natl Acad Sci U S A.

[42] Minter SG (1996). Tenneco: pursuing health for the long term. Occupational Hazards.

[43] Fries JF, Koop CE, Beadle CE, Cooper PP, England MJ, Greaves RF (1993). Reducing health care costs by reducing the need and demand for medical services. N Engl J Med.

[44] Wolfe R, Parker D, Napier N (1994). Employee health management and organizational performance. J Appl Behav Sci.

[45] Stead BA. Worksite health programs: A significant cost-cutting approach. Business Horizons. 1994 1994/11/01:73–8.

[46] Ho JTS (1997). Corporate wellness programmes in Singapore: effect on stress, satisfaction and absenteeism. J Manag Psychol.

